# Interaction studies of *Serendipita indica* and *Zhihengliuella* sp. ISTPL4 and their synergistic role in growth promotion in rice

**DOI:** 10.3389/fpls.2023.1155715

**Published:** 2023-05-24

**Authors:** Neha Sharma, Surbhi Dabral, Jaagriti Tyagi, Gaurav Yadav, Himanshi Aggarwal, Naveen Chandra Joshi, Ajit Varma, Monika Koul, Devendra Kumar Choudhary, Arti Mishra

**Affiliations:** ^1^ Amity Institute of Microbial Technology, Amity University, Uttar Pradesh, India; ^2^ Department of Botany, Hansraj College, University of Delhi, Delhi, India

**Keywords:** microbial interactions, plant growth promotion, *Serendipita indica*, *Zhihengliuella* sp. ISTPL4, *Oryza sativa*, confocal microscopy

## Abstract

Rapid urbanization and globalization demand increasing agricultural productivity. Soil nutrient supply capacity is continuously decreasing due to soil erosion, degradation, salt deposition, undesired element, metal deposition, water scarcity, and an uneven nutrient delivery system. Rice cultivation requires a large amount of water which is becoming detrimental due to these activities. There is a need to increase its productivity. Microbial inoculants are becoming increasingly important in achieving sustainable agricultural production systems. The current study was conducted to investigate the interaction between the root endophytic fungus *Serendipita indica* (*S. indica*) and the actinobacterium *Zhihengliuella* sp. ISTPL4 (Z. sp. ISTPL4) and their synergistic effects on the growth of rice (Oryza sativa L). Both *S. indica* and Z. sp. ISTPL4 showed positive interactions. Growth of S. indica was observed at different days after Z. sp. ISTPL4 inoculation, and stimulated growth of *S. indica* was observed when Z. sp. ISTPL4 was inoculated at 5 dafi (days after fungal inoculation). Z. sp. ISTPL4 promoted the growth of S. indica as it increased spore germination. Furthermore, confocal and scanning electron microscopy (SEM) analyses showed a 27% increase in the spore size of S. indica in the presence of Z. sp. ISTPL4. In a liquid chromatography with tandem mass spectrometry (LC-MS/MS) analysis increased production of alanine and glutamic acid was observed in their sequential co-culture as compared with individual cultures. Sequential inoculation of *S. indica* and Z. sp. ISTPL4 significantly increased the biochemical and physical characteristics of rice as compared with their individual inoculum. Biochemical parameters such as chlorophyll content, total soluble sugar, and flavonoid content in the rice increased by up to 57%, 47%, and 39%, respectively, in the presence of the combined inoculum of *S. indica* and Z. sp. ISTPL4. This will be the first study, to the best of our knowledge, which shows the fungus and actinobacterium interaction and their synergistic roles in the growth promotion of rice. Furthermore, this novel combination can also be used to boost the growth of other crops to increase the agricultural yield.

## Introduction

Rice (*Oryza sativa* L.) is a major agricultural commodity that feeds billions of people worldwide. Future food production will require higher yield and productivity to keep up with the world population’s rapid growth. The production of rice is fraught with difficulties as a result of diseases, weeds, and pests that reduce crop productivity (Sanjeev, 2014).

Farmers regularly utilize a variety of chemical pesticides and fertilizers to boost the yield of their crops, but these fertilizers enrich the soil with secondary nutrients including calcium and sulfur, as well as nitrogen, phosphorus, and potassium, with various negative environmental impacts brought on by these chemical fertilizers (Sharma and Singhvi, 2017). Their higher concentration in soil can affect plant growth negatively by reducing the soil fertility. Furthermore, excessive use of chemical pesticides also affects human health (Sharma and Singhvi, 2017). However, as an alternative, to avoid the adverse impacts of chemical fertilizers and pesticides, biofertilizers can be used. Biofertilizers are microbial-based formulations, i.e., made up of various plant growth-promoting microbes (PGPMs), which help in plant growth and development either directly or indirectly (Naik et al., 2019). The most promising sustainable biostimulants are bacteria, fungi, and algae (Aamir et al., 2020; Abadi et al., 2021; Kaur et al., 2021). These microbes release various chemicals, vitamins, and amino acids, which can be contributing factors to plant growth promotion (Sharma, 2017; Sharma and Singhvi, 2017).

Among various PGPMs, *Serendipita indica* is a well-known root endophytic fungus that was discovered in the Indian Thar desert in 1997 and belongs to the order Sebacinales (Verma et al., 1998). It is well known for its ability to promote plant growth as well as to protect plants from different biotic and abiotic stresses (Tyagi et al., 2017; Lata et al., 2018; Khan et al., 2020; Dabral et al., 2019; Ali et al., 2022). Like fungi, bacteria can also boost plant development either directly or indirectly. They help plants to withstand stress by secreting some antioxidants, including flavanols, flavonoids, and phenolic compounds (Shah and Smith, 2020; Sharma et al., 2021; Bhat et al., 2022; Pathak et al., 2022).

Among bacteria, actinobacteria are Gram-positive, filamentous bacteria that help in plant growth promotion by increasing the area available for absorption and solubilization of phosphate and by organic recycling, and can also serve as bio-inoculants. Soil actinobacteria release certain chemical compounds that promote plant growth (Molina-Romero et al., 2021; Santoyo et al., 2021; Mitra et al., 2022; Tsegaye et al., 2022). Some research has concluded that microbial combinations are more beneficial than their individual inoculations. Although numerous studies have been conducted utilizing *S. indica* alone to observe its plant growth-stimulating capabilities, there have been few studies in which *S. indica* has been combined with other microorganisms (**Table 1**). Dabral et al. (2020) reported a positive interaction between Serendipita indica and Azotobacter vinelandii and also their combined effect on the growth-promoting activities of plants.

**Table 1:** Impact of microbial co-culture on various plant growth.

S. no	Microorganisms	Plant	Response	References
**1.**	*S. indica* + *Z*. sp. ISTPL4	*Oryza sativa*	Co-inoculation of *S. indica* and *Z*. sp. ISTPL4 increased chlorophyll, sugar, carotenoid, and flavonoid content in rice plants	**This study**
**2.**	*S. indica* + *Azotobacter chroococcum* WR5	*Artemisia annua*.	Inoculation of a cell-free supernatant of *Azotobacter chroococcum* WR5 increased glutamate dehydrogenase and attenuated cell wall-degrading enzymes in *S. indica*, resulting in increased spore germination and growth	(Bandyopadhyay, et al., 2016; Arora et al., 2018)
**3.**	*S. indica* + *Pseudomonas striata.*	Chickpea (*Cicer arietinum*)	*Pseudomonas striata* increased the growth of *S. indica*	(Meena et al., 2010)
**4.**	*S. indica* + *Azotobacter vinelandii strain* SRIAz3	Rice (*Oryza sativa*)	*Azotobacter vinelandii* strain SRIAz3 increased growth and spore germination of *S. indica*	(Dabral et al., 2019)
**5.**	*S. indica* and *P. fluorescens*	Chlorophytum sp.	Improved plant growth	(Gosal et al., 2010)
**6.**	*S. indica + Azospirilium brasilense*	-	*Azospirilium brasilense* stimulated *S. indica* growth	(Varma et al., 2012)
**7.**	*S. indica + Bradyrhizobium spp*	*-*	*Bradyrhizobium spp.* stimulated *S. indica* growth	(Ansari et al., 2014)
**8.**	*S. indica + Pseudomonas fluorescens strains WS5*	-	*S. indica* growth is inhibited in the presence of WS5 strain of *Pseudomonas fluorescens*	(Varma et al., 2012)
**9.**	*S. indica + Pseudomonas fluorescens SS101*	-	*Pseudomonas fluorescens* SS101 inhibited the growth of *S. indica*	(Varma et al., 2012)
**10.**	*S. indica+ A. chrococcum M4*	-	*A. chrococcum M4* inhibited the growth of *S. indica*	(Bhuyan et al., 2015)
**11.**	*S. indica + Herbaspirillum frisingense GSF30T*	-	*Herbaspirillum frisingense GSF30T* showed neutral behavior with *S. indica*	(Varma et al., 2012)
**12.**	*S. indica + H.* *lusitanum P6-12*	-	*H. lusitanum P6-12* showed neutral behavior with *S. indica*	(Varma et al., 2012)
**13.**	*S. indica + Bacillus coagulans NCC235*	*-*	*Bacillus coagulans NCC235* showed neutral behavior with *S. indica*	(Varma et al., 2012)
**14.**	*S. indica + Bacillus* *subtilis NCC09*	*-*	*Bacillus subtilis* showed the neutral behavior with *S. indica*	(Varma et al., 2012)
**15.**	*S. indica* *and Pseudomonas sp. R81*	*Solanum lycopersicum*	Enhanced plant biomass	(Sarma et al., 2011)
**16.**	*S. indica + fluorescent pseudomonad strains R62 and R81*	*Vigna mungo*	Increased yield of *V. mungo* due to improved nutrient uptake	(Kumar et al., 2011)

Until now, the interaction between *S. indica* and actinobacteria has not been studied. Therefore, it would be interesting to investigate the combined interactions among fungi and actinobacteria and their symbiotic effects on the growth of rice. This is the first research that will use the novel combination of *S. indica* and *Zhihengliuella* sp. ISTPL4 in documenting plant growth promotion activity as well as alterations in the symbiont morphology. Until now, no research has been conducted on *Z.* sp. ISTPL4, a Gram-positive actinobacterium that was isolated from Pangong Lake, Ladakh, Jammu, and Kashmir, India (Mishra et al., 2020). In the current research, it is concluded that the genus *Zhihengliuella* acts as a plant growth promoter even under high-salt stress conditions (Siddikee et al., 2010; Paul and Lade, 2014). We hypothesize that this novel combination of *S. indica* and *Z.* sp. ISTPL4 will be beneficial for plant growth promotion, and that it may be used for the mitigation of other abiotic stresses.

## Materials and methods

### Microbial strains and culture conditions

#### Serendipita indica

Growth of *S. indica* was maintained in the Hill and Kafer (HK) media and broth by culturing a 4-mm disc of *S. indica* and then kept for incubation in the dark for 2 weeks at 28–30°C (centrifuged at 120 rpm in case of broth) (Hill and Kafer, 2001).

#### *Zhihengliuella* sp. ISTPL4

The culture of *Z.* sp. ISTPL4 was maintained in Luria–Bertani (LB) agar at 28–30°C for a period of 24 hours. After 24 hours of incubation, a single colony of *Z.* sp. ISTPL4 was picked and inoculated in Luria broth following incubation at 28–30°C at 120 rpm for 24 hours. A 100-µL inoculum from this primary culture was added into fresh LB broth and maintained at the same conditions. This culture is termed a secondary culture. This secondary growth culture of *Z.* sp. ISTPL4 was centrifuged at 10,000 rpm for 5 minutes, followed by the preparation of a cell suspension to a concentration of 400 cfu/mL (optical density (OD) of 0.6).

#### Co-culture of *S. indica* and *Z.* sp. ISTPL4

The co-culture of *S. indica* and *Z*. sp. ISTPL4 was prepared by adding 1 mL of the secondary culture *Z*. sp. ISTPL4 with an OD of 0.6 at 5 dafi. The co-culture was incubated at 28–30°C for 2 weeks, with shaking at 120 rpm. The freshly grown individual culture, as well as the co-culture, was then centrifuged at 10,000 rpm for 10 minutes. The resulting supernatant was lyophilized and the lyophilized powder used for further analysis.

### Effect of *Z.* sp. ISTPL4 on the growth of *S. indica*

The methodology for interaction studies between *S. indica* and *Z*. sp. ISTPL4 was adapted from Khumar Bhuyan et al. (2015), with modifications in the culture medium used. For interaction studies, mixture of Hill and Kaefer with Luria Bertani agar in a ratio of 1:3 was used. In this study, a fungal disc from a freshly grown culture of S. indica measuring up to 4 mm was inoculated. The culture plate was then incubated at 28°C–30°C for 3 and 5 days, respectively. A secondary culture of Z. sp. ISTPL4 was streaked toward the periphery on days 3 and 5 after fungal inoculation. Further growth of S. indica was measured in terms of the increase in hyphal growth radius. To check the interaction in broth, liquid broth was used, and dry cell weights were determined. A 4-mm fungal disc was added to 50 mL of a mixture of Hill and Kaefer with Luria Bertani agar (1:3) and then incubated at 28–30°C, with shaking at 120 rpm. After 5 days of incubation, 1 mL of the secondary culture of Z. sp. ISTPL4 was added and the culture again incubated. This experiment was carried out for 15 days to observe the effect of Z. sp. ISTPL4 on the biomass of fungus. Each experiment was performed in five sets. 

#### *S. indica* spore morphology in the presence of *Z.* sp. ISTPL4

The methodology used for observing the influence of the cell-free supernatant of *Z.* sp. ISTPL4 on the spores of *S. indica* was taken from Bandyopadhyay et al. (2016). For this, a bacterial culture of *Z*. sp. ISTPL4 was grown in a LB medium and added to HK minimal medium for 96 hours at 28°C–30°C, with shaking at 120 rpm, followed by centrifugation of bacterial culture at 10,000 rpm to obtain a cell-free supernatant (Labogene, Denmark). The resulting cell-free supernatant was filtered *via* a 0.22-µm membrane (Millipore) and then inoculated in fungal spores (4.85 × 10^5^ spores/mL) for an incubation period of 12 hours. The spore morphology of *S. indica* was observed under a Nikon confocal microscope (model Nikon A1) at 60× magnification.

#### *S. indica* spore size in presence of *Z.* sp. ISTPL4

The effect of *Z*. sp. ISTPL4 on the spore size of *S. indica* was analyzed by a compound microscope, scanning electron microscopy, and confocal microscopy. *S. indica* spores were taken from the control as well as from the co-cultured plate. A small colony from a fungal mat was picked up and gently placed on a glass slide followed by teasing with the help of needles. It was then further stained with lactophenol cotton blue (LPCB). The slide was then kept for 5 minutes at room temperature. Spores of *S. indica* were observed at 40× using a Nikon compound microscope. For the SEM analysis, sections of fungal discs from control plates as well as interaction plates were fixed in 2.5% glutaraldehyde and incubated for 1 hour at room temperature. Following incubation at 4°C overnight, samples were centrifuged at 5,000 rpm for 5 minutes (Sangster et al., 2006). Subsequently, the samples were washed with a 0.1 M phosphate buffer (pH 7, 5 minutes) then centrifuged at 5,000 rpm (5 minutes). Pellets were then dissolved in 0.1% filter-sterilized silver nitrate (AgNO_3_) solution and incubated for 1 hour at room temperature. Samples were then gradually dehydrated in graded series of ethanol (30%–90%) for 15 minutes (Gaba et al., 2022). The final step was performed thrice in 100% ethanol for 5 minutes each. The samples were then observed under the SEM. The samples were dehydrated, air dried, and placed on double-adhesive carbon conductive tape in aluminum specimens, and then gold coated in a Quorum Q150ES coater 23 for 1 minute. The samples were then examined using a SEM (model Zeiss EVO 18; Zeiss, Raipur, India).

Confocal microscopy was used to inspect the structural and size modifications in the *S. indica* spores. Samples were prepared as described by Hilbert et al. (2013). Spores were collected by adding 1 ml of autoclaved 0.02% Tween 20 solution and gently scraping the freshly grown *S. indica* culture from both control and interaction plates. This was followed by centrifugation for 10–15 minutes at 5,000 rpm. The supernatant was discarded, and the pellet was mixed with 10 mL of autoclaved distilled water. Spore count was determined and adjusted to a concentration of 4.85 × 10^5^ spores/mL. Spore size was determined using NIS Elements software (Nikon, Japan) and a Nikon confocal microscope A1 at 60× magnification at the Amity Institute of Microbial Technology, Amity University, India (Dabral et al., 2020).

### Amino acids analysis of *S. indica*, *Z.* sp. ISTPL4, and their co-culture

Liquid chromatography coupled with SCIEX 6500 and triple quadruple-trap MS/MS (LC-MS/MS) was used to analyze the amino acids produced by the individual cultures of *S. indica* and *Z.* sp. ISTPL4 as well as their co-inoculation. Sample preparation was done by taking 25 mg of lyophilized powder (containing bacterial and fungal sample each) was measured and mixed with 1 ml of 80% methyl alcohol. The methanolic extract was then diluted in water containing a ^13^C- and ^15^N-labeled algal amino acids mix in a 1:20 (v:v) ratio (Cambridge Isotope Laboratories, Inc., USA). The material was subsequently separated into amino acids using a Zorbax Eclipse XDB-C18 column (50 x 4.6 mm, 1.8 m, Agilant Technologies) (Zelena et al., 2009; Dunn et al., 2011). The composition of mobile phase A comprised 0.1% formic acid and water and that of mobile phase B comprised 0.1% formic acid and acetonitrile. The protocol for gradient elution comprised 0–0.35 minutes, solution B (2%); 0.35–13.4 minutes, solution B (2%–57%); 13.4–15.7 minutes, solution B (57%–100%); and 15.8–18 minutes, solution B (100%–2%) (Sangster et al., 2006). Other parameters of chromatography included temperature 27°C, constant flow of 1.5 mL/min, total run time of 18 minutes, and a derivation reagent that included 9-fluorenylmethyl chloroformate and *o*-phthalaldehyde.

### Rice growth conditions

Seeds of *Oryza sativa* (Taipei 309) were obtained from the National Institute of Plant Genome Research, New Delhi. The soil used in this study had a composition of sandy loam with a pH 7.4, and was autoclaved three times at 121°C at a pressure of 15 psi before use. The pot experiment was designed for pots that were 11 cm in height and 9 cm in diameter and had a capacity of 265 g of soil. The composition of the soil was peat and soil in a ratio of 1:2. Four seeds were added to each pot and then kept for seed germination for 7–10 days. Each experimental set-up was carried out in five sets. There were four pots in each set (control; plants treated with *S. indica* only; plants treated with Z. sp. ISTPL4 only; and plants treated with a combination of *S. indica* and *Z.* sp. ISTPL4) and a total of 20 pots in each experimental set-up. Half-strength Hoagland’s solution (40 mL) was added to the seedlings twice per week (Hoagland and Arnon, 1950).

### Inoculation of *S. indica* and *Z* sp. ISTPL4 in rice

Spore isolation and spore count of *S. indica* were determined using the Fuchs–Rosenthal counting chamber and maintained to a final concentration of 4.85 × 10^5^ spores/mL before inoculating the plant. Similarly, a fresh culture of *Z.* sp. ISTPL4 was centrifuged at 7,000 rpm for 20 minutes and adjusted to 0.6 OD (Kilam et al., 2015). After 1 week of seed germination, the rhizospheric regions of the seedlings were pierced, and inoculated with a culture of *S. indica* containing 4.85 × 10^5^ spores/mL. Subsequently, at 5 dafi, plants were inoculated with 1.0 mL of *Z.* sp. ISTPL4 culture. Plants cultures of *S. indica* and *Z.* sp. ISTPL4 were also inoculated individually.

### Morphological parameters of plant

Various physical characteristics, including shoot length, root length, number of leaves and lateral roots, root fresh/dry weight, and shoot fresh/dry weight, were observed. All the experiments were carried out in triplicate.

### Biochemical parameters of plants

#### Total chlorophyll content

Leaf samples were crushed in 80% acetone (v/v) and then stored in the dark for 24 hours. Using a UV–Vis spectrophotometer (Labogene, Denmark), absorbance measurements at 645 and 663 nm were taken and the total chlorophyll content was calculated as mg/g fresh weight (FW) using Arnon’s formula (Arnon, 1949).

#### Total soluble sugar

Total soluble sugar levels were calculated using the anthrone method: a leaf sample weighing 0.5 g was crushed in 80% ethanol and centrifuged at 8,000 rpm for 10 minutes. The supernatant was combined with 4 mL of freshly made anthrone sulfuric acid solution (100 mL of 75% sulfuric acid, w/w, and 150 mg of anthrone), and then incubated for 10 minutes in a boiling water bath (50°C) (Gengmao et al., 2014). Finally, absorbance was measured at 620 nm by a spectrophotometer. Glucose equivalent was used to express the amount of soluble sugar (mg/g FW of sample).

#### Flavonoids content

The aluminum chloride colorimetric method was used to measure the total flavonoid content (Chang et al., 2002). This was carried out by adding 1.0 g of leaf sample to 10 mL of methanol. After centrifugation at 12,000 rpm for 20 minutes, a 0.5-mL sample of methanolic extract was obtained and homogenized with 0.1 mL of a 10% solution of aluminum chloride, 0.1 mL of 1 M potassium acetate, 1.5 mL of methanol, and 2.8 mL of distilled water. The resulting mixture was then maintained at room temperature for 30 minutes before measuring absorbance at 415 nm with a spectrophotometer. Using quercetin as the standard solution, a calibration curve was used to determine the total flavonoids concentration. Values were given as percentage of the dry weight of quercetin equivalent per gram (Arora et al., 2020).

### Statistical analysis

All the tests were repeated, and the data shown are the averages of at least five replicates. The means, standard deviation, and graphs were computed using Microsoft Excel 2007. Statistical data were analyzed by two-way ANOVA and Student’s *t*-test.

## Results

### Interaction of *S. indica* and *Z.* sp. ISTPL4

**Figure 1:**
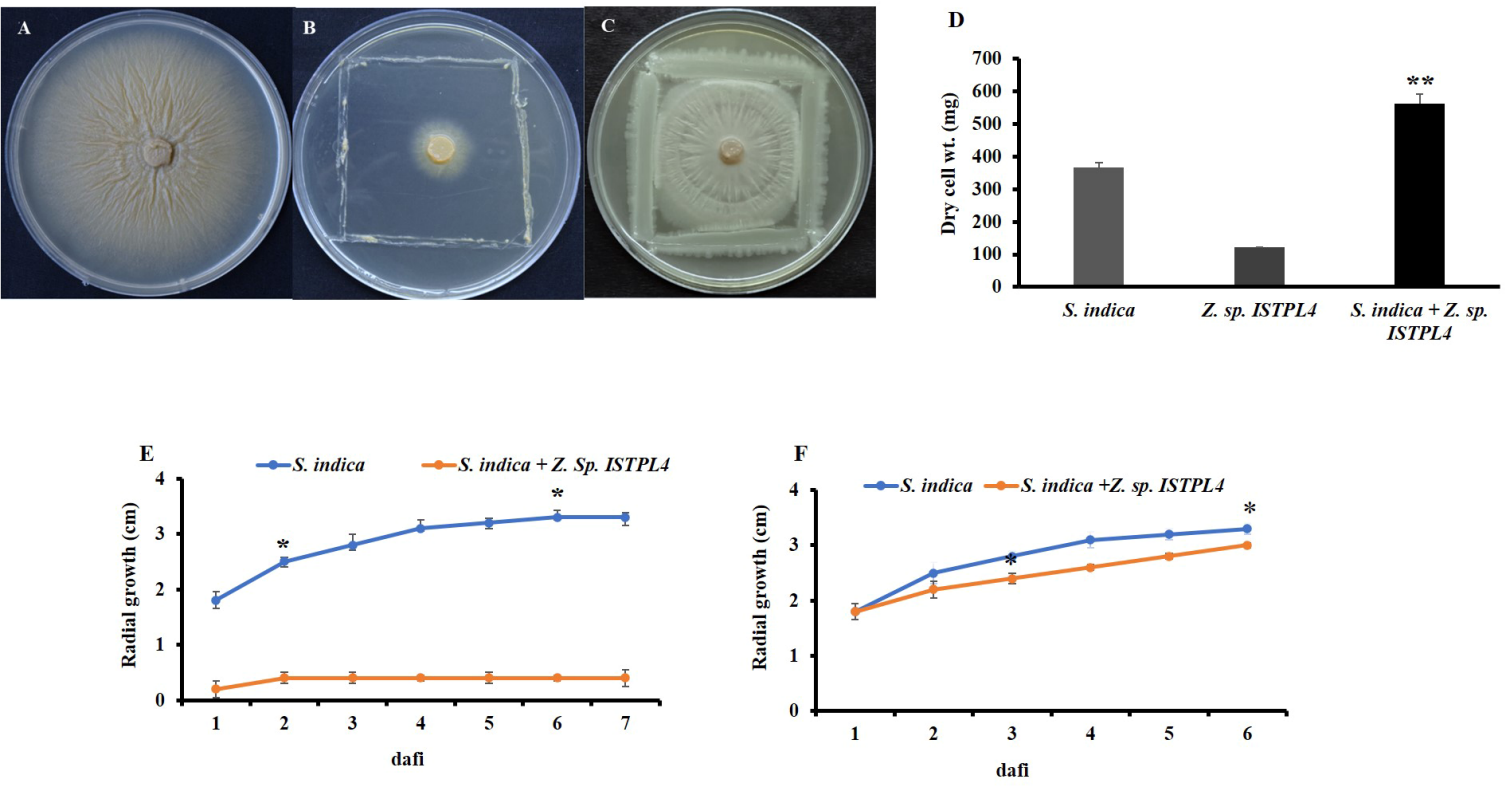
Interaction analysis of S. indica and Z. sp. ISTPL4 **(A)** growth of S. indica without Z. sp. ISTPL4; Control **(B)** growth of S. indica in the presence of Z. sp. ISTPL4 at 3 days after fungal inoculation (3 dafi) **(C)** growth of S .indica in presence of Z. sp. ISTPL4 at 5 days after fungal inoculation (5 dafi) **(D)** increasing dry weight of S. indica alone and in presence of Z. sp. ISTPL4 **(E)** increasing mycelial radii growth of S. indica at 3 dafi **(F)** represent increasing mycelial radii of S. indica at 5 dafi; Values are means of five biological replicates and standard errors are indicated with P<0.05; DAFI: days after fungal inoculation. According to the student’s t-test, the asterisks showed significant differences. ‘*’: P ≤ 0.05; ‘**’: P ≤ 0.01; ‘***’: P ≤ 0.001.

The effect of *Z*. sp. ISTPL4 on the growth of *S. indica* on agar plates was determined from the increase in the radial growth of fungus compared with the control plate. Sequential inoculation with *Z.* sp. ISTPL4 at 3 dafi resulted in limited fungal growth (1.3 ± 0.04 cm; control 3.3 ± 0.02 cm), whereas the sequential inoculation of *Z.* sp. ISTPL4 at 5 dafi resulted in stimulated fungal growth (2.9 ± 0.06 cm; control 3.3 ± 0.02 cm) (**Figures 1A–F**).

### Effect of *Z.* sp. ISTPL4 on biomass of *S. indica*

There was a 23% increase in the dry cell weight of the combined culture of *S. indica* and *Z.* sp. ISTPL4 compared with their respective controls (**Figure 1D**).

### Impact of *Z.* sp. ISTPL4 on cell free supernatant of *S. indica*

**Figure 2:**
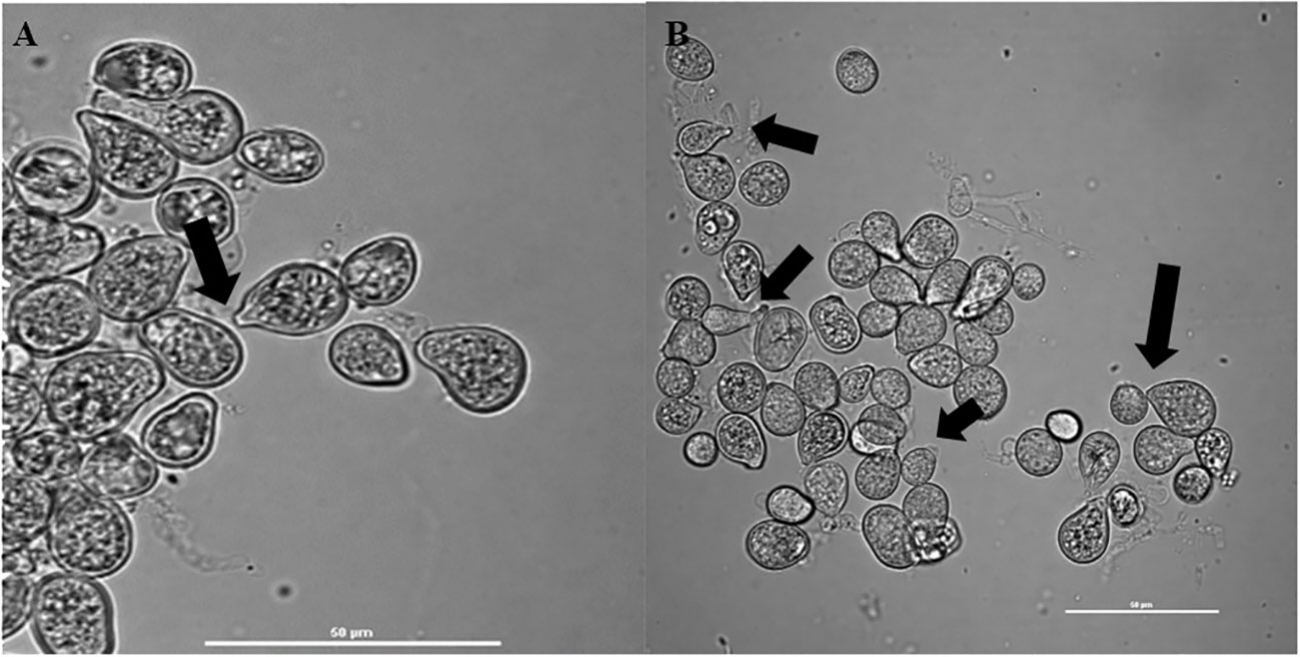
Confocal microscopic analysis shows spore germination of **(A)** S. indica alone in Hill and Kaefer minimal media and **(B)** in the presence of Z. sp. ISTPL4, arrow indicating hyphae of germinating spores.

Confocal microscopic examination showed that the cell-free supernatant promoted spore germination of fungus (**Figures 2A, B**). Additionally, analyses of spore germination revealed that the presence of cell-free supernatants of bacteria promoted spore germination in fungus when compared with the control.

### Effect on spore size of *S. indica* in presence of *Z.* sp. ISTPL4

**Figure 3:**
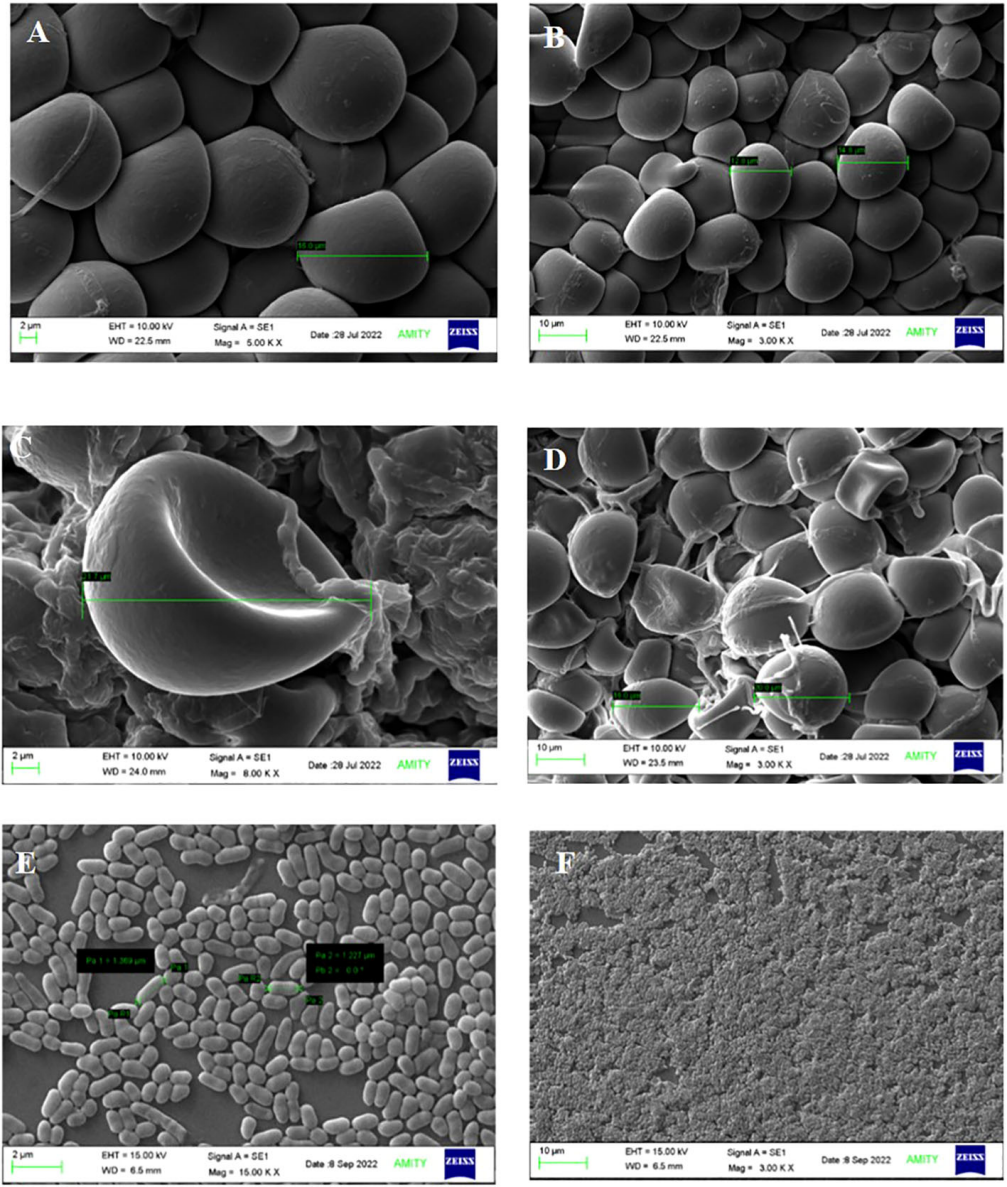
Scanning Electron Microscopic analysis shows the spore morphology **(A, B)** S. indica alone **(C, D)** in the presence of Z. sp. ISTPL4 **(E, F)** cell structure of Z. sp. ISTPL4.

**Figure 4:**
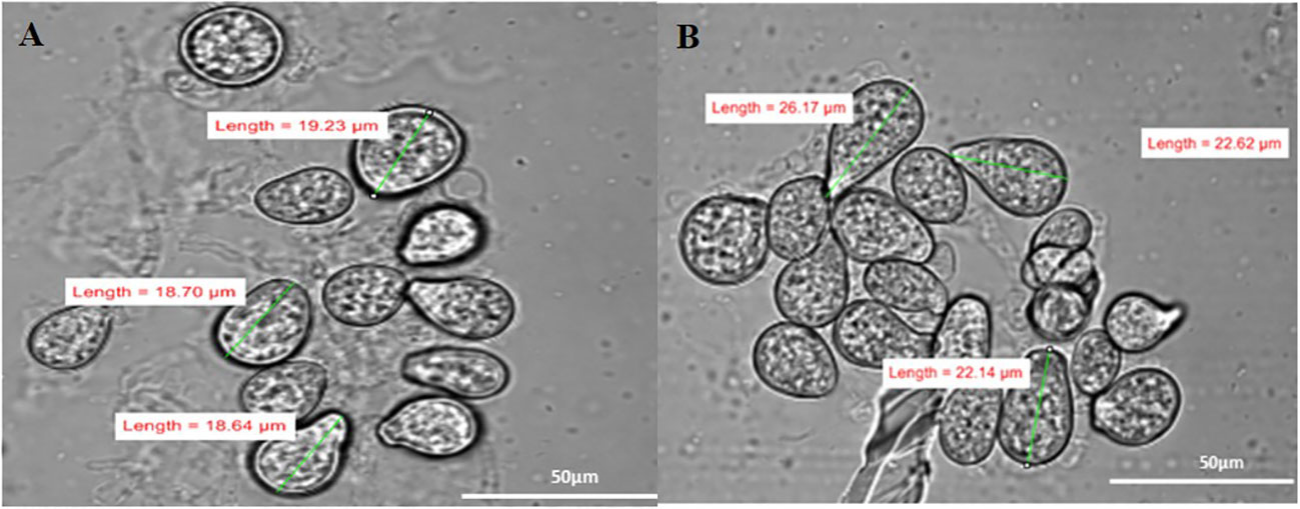
Confocal microscopy analysis shows the spore size of **(A)** S. indica alone **(B)** in the presence of Z. sp. ISTPL4.

The spore size of *S. indica* increased in the presence of *Z.* sp. ISTPL4, as observed using a Nikon compound microscope (**Supplementary Figure 1**). SEM and confocal microscopic analyses revealed that there was a 27% increase in the fungal spores in the presence of *Z.* sp. ISTPL4 (**Figures 3A–F**, **4A, B**).

### Amino acid yield in individual cultures of *S. indica*, *Z.* sp. ISTPL4s, and their co-cultures

Data from the amino acid analyses revealed the production of various amino acids. Eleven amino acids which were found to be produced at higher levels in the sequential co-culture (5 dafi) of *S. indica* and *Z.* sp. ISTPL4 than in the individual cultures. These were alanine, valine, proline, threonine, isoleucine, glutamic acid, histidine, phenylalanine, arginine, tyrosine, and tryptophan. Three amino acids, serine, glutamine, and lysine, were produced at lower levels in the sequential co-culture of *S. indica* and *Z.* sp. ISTPL4 than in the individual cultures. The concentrations of some amino acids, such as alanine and glutamic acid, were higher in the sequential co-culture of *S. indica* and *Z.* sp. ISTPL4 than in the individual cultures. The concentration of alanine was 25.48 µM in the sequential co-culture, whereas it was 11.406 µM and 30.9 µM in individual cultures of *S. indica* and *Z.* sp. ISTPL4, respectively. Similarly, the concentration of glutamic acid was 11.511 µM in the sequential co-culture of *S. indica* and *Z.* sp. ISTPL4, which was approximately half in individual culture of *S. indica*. The concentrations of the amino acids serine, glutamine, and lysine were 0.7, 3.4, and 6.8 µM, respectively, in the sequential co-culture of *S. indica* and *Z.* sp. ISTPL4, compared with 1.06, 3.5, and 6.6 µM, respectively, in the individual culture of *Z*. sp. ISTPL4 and 0.5, 2.3, and 5.1 µM, respectively, in the individual culture *S. indica*, (**Supplementary Tables 1A, B**).

### Effect on the morphological parameters of rice

**Figure 5:**
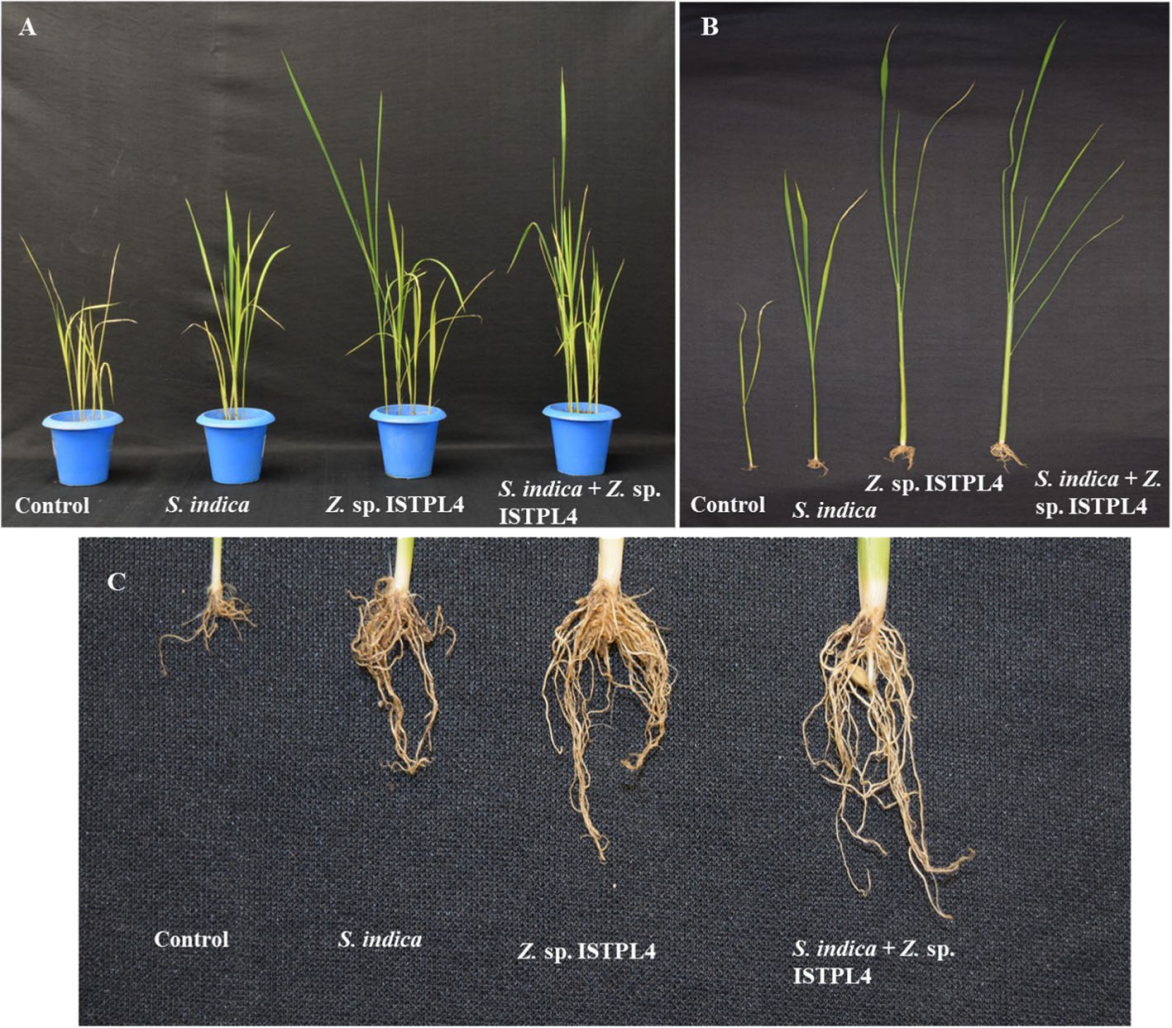
Effect of **(A)** individual culture of *S. indica* and Z. sp. ISTPL4 alone and in the presence of Z. sp. ISTPL4 on the growth-promoting activities of rice (Taipei 309) **(B)** represents the difference in shoot length of plants treated with individual inoculation of *S. indica* and Z. sp. ISTPL4 alone and sequential inoculation *S. indica* and Z. sp. ISTPL4 **(C)** represent the difference in root length of plants treated with individual inoculation of *S. indica* and Z. sp. ISTPL4 alone and sequential inoculation *S. indica* and Z. sp. ISTPL4.

**Figure 6:**
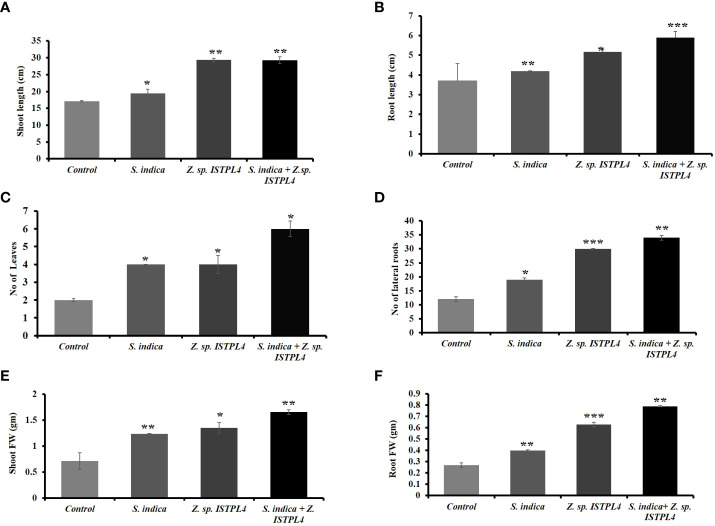
**(A)** represents the shoot length **(B)** root length **(C)** the number of leaves and **(D)** number of lateral roots **(E)** shoot fresh/dry weight **(F)** root fresh/ dry weight of rice treated with individual inoculation of S. indica and Z. sp. ISTPL4 respectively and sequential inoculation S. indica and Z. sp. ISTPL4; Values are means of five biological replicates with standard error with P < 0.05; DAFI: days after fungal inoculation. According to the student’s t-test, the asterisks showed significant differences. ‘*’: P ≤ 0.05; ‘**’: P ≤ 0.01; ‘***’: P ≤ 0.001.

Various physical parameters, such as root length, shoot length, numbers of leaves and lateral roots, shoot fresh and dry weight, and root fresh and dry weight, were recorded (**Figures 5A–C**, **6A–F**). Overall, plant growth was found to be highest in plants that were treated with sequential inoculation of *S. indica* and *Z.* sp. ISTPL4. Shoot length increased by 41%, 11%, and 41.6%, respectively, in plants treated with sequential *S. indica* and *Z.* sp. ISTPL4 inoculation, individual *S. indica* inoculation, and individual *Z.* sp. ISTPL4 inoculation.

Plant root length increased by 37%, 11%, and 27%, respectively, in plants treated with sequential inoculation of *S. indica* and *Z.* sp. ISTPL4, individual *S. indica* inoculation, and individual *Z.* sp. ISTPL4 inoculation.

The numbers of leaves and lateral roots were also increased in plants treated with sequential inoculation of *S. indica* and *Z*. sp. ISTPL4 followed by in plants treated with individual inoculation of Z. sp. ISTPL4 or *S. indica* (**Figures 6C, D**). The number of lateral roots observed in plants treated with sequential inoculation of *S. indica* and *Z.* sp. ISTPL4 and individual inoculation of *Z.* sp. ISTPL4 and *S. indica* was, respectively, 64%, 60%, and 36%, higher than in control plants, while the number of leaves was, respectively, 66%, 50%, and 50% higher.

The fresh and dry weight of roots were, respectively, 65% and 35% higher in plants that were treated with sequential inoculation of *S. indica* and *Z.* sp. ISTPL4 than in control plants. Similarly, the fresh and dry weight of shoot samples were, respectively, 48% and 31% higher in plants treated with sequential inoculation of *S. indica* and *Z*. sp. ISTPL4 than in control plants.

### Effect of *S. indica*, *Z*. sp. ISTPL4, and their co-culture on rice

**Figure 7:**
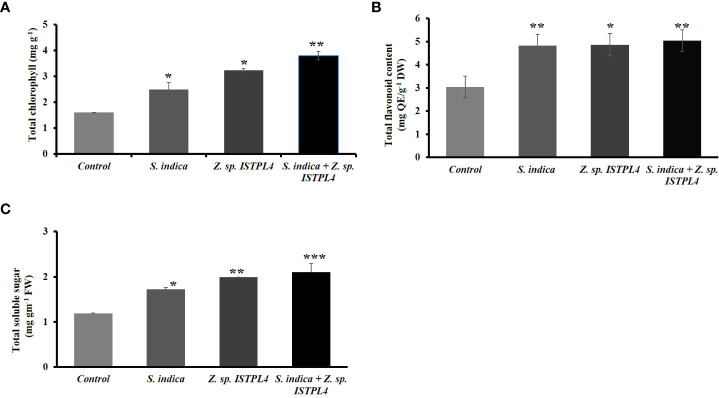
Estimation of various biochemical parameters including **(A)** Total chlorophyll content **(B)** Total Flavonoids content and **(C)** Total soluble sugar of plants treated with individual inoculation of S. indica and Z. sp. ISTPL4 alone and sequential inoculation S. indica and Z. sp. ISTPL4; Values are means of five biological replicates and standard errors are indicated with P<0.05; DAFI: days after fungal inoculation. According to the student’s t-test, the asterisks showed significant differences. ‘*’: P ≤ 0.05; ‘**’: P ≤ 0.01; ‘***’: P ≤ 0.001.

Biochemical parameters such as chlorophyll content, flavonoid content, and total soluble sugar content were also measured (**Figures 7A–C**). Levels were highest in plants treated with sequential inoculation of *S. indica* and *Z.* sp. ISTPL4, followed by plants treated with individual inoculation of *Z.* sp. ISTPL4 and plants treated with individual inoculation of *S. indica*. Chlorophyll content was measured in terms of mg/g fresh weight. It was increased more in plants that were treated with sequential inoculation of *Z.* sp. ISTPL4 and *S. indica* (57%) than in plants treated with individual inoculation of *Z.* sp. ISTPL4 (50%) or *S. indica* (36). Total soluble sugar was measured in terms of mg/g. It too was increased more in plants that were treated with sequential inoculation of *Z.* sp. ISTPL4 and *S. indica* (47%) than in plants treated with individual inoculation of *Z.* sp. ISTPL4 or *S. indica* (42% and 35%, respectively). Flavonoid content was estimated in terms of mg Quercetin/g. It was increased in all treated plants, but to a slightly greater degree in plants that were treated with sequential inoculation of *S. indica* and *Z.* sp. ISTPL4 (39%) than in plants treated with individual inoculation of *Z.* sp. ISTPL4 or *S. indica* (37% and 36%, respectively).

## Discussion

Microorganisms play an important role in promoting plant growth and increasing agriculture productivity. They help in nutrient recycling and increase the absorption area of roots, thus increasing the mobilization of various nutrients, such as phosphorus, potassium, and nitrogen. They also promote plant growth, and protect plants from various biotic and abiotic stresses (Yadav et al., 2021). According to Dabral et al. (2020), the combined culture of *S. indica* and *SRIAz3* at 7 dafi stimulated growth of *S. indica* while it inhibited growth of *S. indica* at 3 dafi (Dabral et al., 2020). In another study, Kumar Bhuyan et al. (2015) found an increase in growth of *S. indica* under the positive influence of *Azotobacter chroococcum* WR5 after 6 days of interaction (Kumar Bhuyan et al., 2015). In support of our results, *in vitro* interaction study revealed growth stimulation at 5 dafi has been observed. Simultaneous and sequential inoculation of fungus and bacterium have varied effects on fungal growth. Unique bacterial metabolites produced in the environment during co-cultivation may be responsible for fungal growth stimulation. There was an increase in the dry cell weight of *S. indica* in the presence of *Z.* sp. ISTPL4 as compared with their individual inoculation. According to Kumar Bhuyan et al. (2015), the dry cell weight of *S. indica* co-cultured with *A. chroococcum* WR5 was substantially greater than that of *S. indica* cultured alone (Kumar Bhuyan et al., 2015). Our findings are consistent with their findings.

To observe the influence of a cell-free supernatant of bacteria on fungal spore germination, we studied spore germination in the presence of *Z.* sp. ISTPL4. The increased germination of fungal spores was observed in the presence of *Z.* sp. ISTPL4, suggesting that the presence of active bacterial metabolites may be responsible for fungal growth promotion. Our observations are similar to those reported by Bandyopadhyay et al. (2016), who demonstrated that diffusible components from *A. chroococcum* altered the pattern of spore germination (Bandyopadhyay et al., 2016). Increased spore germination has been linked to the stimulation of fungal glutamate dehydrogenase and the attenuation of cell wall-degrading enzymes (α-glucoside b) in the presence of WR5.

The results of SEM also revealed an increased spore size of *S. indica* grown in the presence of *Z.* sp. ISTPL4 compared with *S. indica* alone. Some bacteria have been shown to directly affect Arbuscular mycorrhizal fung (AMF) fungal growth and germination (Artursson et al., 2006; Dabral et al., 2020). Kumar Bhuyan et al. (2015) also reported the thickness of fungal hyphae wall and the formation of intermittent globular structures in clusters as well as in isolation in the presence of strain WR5. In interactions between microbes and plants, secretory proteins are the main actors (De-la-Peña et al., 2008; Netzker et al., 2015; van Dam and Bouwmeester, 2016). The physiology of fungi is impacted by the bacterial secretion system, which promotes or limits the growth of fungi. Similarly, sequential inoculation of the fungus *Vibrio vulnificus* results in increased production of bioactive compounds, secondary metabolites, and various amino acids (Miao et al., 2006).

Amino acids play a crucial role in microbial interactions as well as in plant growth. They act as signaling molecules in various metabolic cascades and help in cell-to-cell communication (Frey-Klett et al., 2011; Poveda et al., 2022). The analysis of various amino acids was done using LC-MS/MS. Kumar Bhuyan et al. (2015) reported an increased expression of several important metabolic proteins when P. indica was co-cultured with WR5. According to various reports, there was a significantly higher concentration of five amino acids including homoserine, alanine, aspartic acid, methionine, and isoleucine in the individual culture of Bacillus amyloliquefaciens while the concentration of asparagine and serine were higher in the individual culture of Trichoderma asperellum (T). Histidine, lysine, phenylalanine, proline, tryptophan, and tyrosine were higher in simultaneous co-culture of TB1 (T. asperellum and B. amyloliquefaciens co-culture), while glutamine and aspartic acid were increased in sequential co-culture of TB2 (sequential inoculation-based T. asperellum and B. amyloliquefaciens co-culture) (Karuppiah et al., 2019). Similarly, eight amino acids, including l-allothreonine, d-aspartic acid, l-glutamic acid, l-histidine, l-serine, l-leucine, l-isoleucine, and l-proline were detected in BT1 (Co-culture of Bacillus amyloliquefaciens ACCC111060 and Trichoderma asperellum GDFS1009) in significantly high amounts (Wu et al., 2018). In our study, 14 different amino acids were detected through LC-MS/MS in the individual culture of *S. indica* and *Z.* sp. ISTPL4 as well as their sequential co-culture. Concentrations of alanine, valine, proline, threonine, isoleucine, glutamine, histidine, phenylalanine, arginine, tyrosine, and tryptophan were higher in the sequential co-culture of *S. indica* and *Z.* sp. ISTPL4. Concentrations of glutamic acid and alanine were higher in the individual culture of *Z*. sp. ISTPL4, whereas lower concentrations of amino acids such as serine, glutamine, and threonine were found in the sequential co-culture of *S. indica* and *Z.* sp. ISTPL4, followed by the individual culture of *S. indica* alone. These amino acids play a crucial role in the growth-promoting activities of plants (Fujita et al., 2006; Singh et al., 2017). Similar outcomes were reported in another study in which the interaction of *S. indica* with *A. chroococcum* resulted in an increased production of alanine while the concentration of glutamine and glutamic acid decreased. In fungi, alanine plays a vital role. It act as an end product for the glucose carbon and a convenient reservoir of both pyruvate and amino groups in mycelia during periods of sufficient carbon and nitrogen supply (Leyva-Rojas et al., 2020). However, glutamic acid is an important amino acid. It is crucial for the growth and development of plants. It helps in increasing the photosynthetic yield by synthesizing chlorophyll content and also aids in nitrogen assimilation by converting ammonium into glutamate and glutamine in plants (Bernard and Habash, 2009). Some amino acids, for example alanine, play a crucial role in the growth of plants and help plants to withstand various stresses, such as waterlogging, drought, etc. (Netzker et al., 2015). Other amino acids, such as valine, threonine, and lysine, are essential amino acids and were more elevated in the sequential co-culture of *S. indica* and *Z*. sp. ISTPL4 than in their individual culture. Increased concentrations of these amino acids clearly indicate a role for these amino acids in plant growth promotion activities.

In general, plant–microorganism interactions in the rhizospheric region are critical for nutrient uptake, stress alleviation, and plant development (Ali et al., 2022). The inoculation and introduction of beneficial bacteria into plants has long been regarded as the most important method of improving plant health and productivity (Zhang et al., 2022). This study examined the effects of *S. indica* and *Z.* sp. ISTPL4 on rice (Taipei 309) plants cultivated *in vitro*, either individually or in combination. The results showed that growth of shoots and roots was positively impacted by microbial inoculants. Overall, compared with single-inoculated *S. indica* or *Z.* sp. ISTPL4 plants, the dual-inoculated (*S. indica* + *Z.* sp. ISTPL4) plantlets consistently produced the best outcomes. Our findings are the consistent with the results of Arora et al. (2016): they also observed enhanced biomass, as well as increased plant roots and shoots, in artemisinin that was inoculated with *P. indica* and *A. chroococcum* individually and in combination. In the current study, plants co-inoculated with *S. indica* and *Z.* sp. ISTPL4, root length, shoot length, plant biomass ratio, and the numbers of leaves and lateral roots were all increased. Biochemical parameters, such as total soluble sugar content, were found to be higher in plants treated with sequential inoculation of *S. indica* and *Z.* sp. ISTPL4 than in plants treated with individual inoculation of *S. indica* or *Z.* sp. ISTPL4. Endophytic fungus can potentially influence plant growth by directly influencing hormonal pathways.

Asim et al. (2022) observed increased root and aerial biomass, root and stem length, vigor index, and germination using wheat (*Triticum aestivum*) and endophytic fungus *Fusarium oxysporum* (Asim et al., 2022; Poveda et al., 2022). It has also been reported that inoculating plants with *S. indica* or Arbuscular mycorrhizal fungi (AMF) boosts photosynthetic efficiency by increasing the chlorophyll content of leaves, surface area of leaves, stomatal conductance, and sugar content of leaves (Das et al., 2012; Baishya et al., 2015). Gosal et al. (2010) observed that *S. indica*-inoculated plants had a higher chlorophyll content than non-inoculated plants (Gosal et al., 2010). Our study also revealed similar results, including higher total chlorophyll content, sugar content, and flavonoid content in plants treated with the sequential inoculation of *S. indica* and *Z*. sp. ISTPL4 than in plants treated with individual inoculation of *S. indica* or *Z.* sp. ISTPL4 alone. The increased chlorophyll content, sugar content, and flavonoid content help plants to cope with environmental stresses.

Microbial interactions are crucial for promoting plant growth in the rhizospheric soil. A combination of *S. indica*, *A. chroococcum*, and *Rhizophagus intraradices*, increased the growth of *Zea mays* by increasing shoots, roots, and biomass accumulation (Tyagi et al., 2023). Co-inoculation of *S. indica* and *A. chroococcum* has been shown to boost *Stevia rebaudiana* growth by enhancing photosynthetic molecule biosynthesis, resulting in increased chlorophyll, carotenoids, total sugar, and protein levels (Kumar Bhuyan et al., 2015). Co-inoculation of *S. indica* and PGPB (*Enterobacter asburiae*, *Lactococcus lactis*, and *A. chroococcum*) improved the growth of *Trigonella foenum-graecum* by increasing the photosynthetic yield, transpiration rate, stomatal conductance, and internal CO_2_ (Bisht et al., 2022). Plant growth-promoting bacteria help in the synthesis of phytohormones such as auxin, which is involved in coleoptile elongation during submergence (Ahumada et al., 2022). This work is novel and suggests the synergistic effect of microbes (*S. indica* and *Z.* sp. ISTPL4) in plant growth promotion activities. This strong promotion of plant growth due to the sequential inoculation of *S. indica* and *Z.* sp. ISTPL4 constitutes a new finding that will help increase sustainable agricultural practices and improve crop productivity.

## 5 Conclusion

In this study, we observed that the sequential inoculation of Z. sp. ISTPL4 at 5 dafi stimulated the growth of S. indica. In comparison with individual inoculation of either fungus or bacterium, sequential inoculation of Z. sp. ISTPL4 promoted plant growth in terms of increased plant height, shoot length, root length, number of leaves, and number of lateral roots. A significant increase in photosynthetic pigments, sugar content, and flavonoid content was also observed in plants that were sequentially inoculated with Z. sp. ISTPL4. This finding suggests better plant growth after sequential inoculation of microbes for better agriculture sustainability. This combination of microbes will be beneficial for field studies. There are various documented reports of the use of S. indica to mitigate various biotic and abiotic stresses, while Z. sp. ISTPL4 has been reportedly used in heavy metal remediation. Therefore, it might be beneficial to use a combination of S. indica and Z. sp. ISTPL4 for plant growth promotion and to ameliorate abiotic stress.

## Data availability statement

The original contributions presented in the study are included in the article/**Supplementary Material**. Further inquiries can be directed to the corresponding authors.

## Author contributions

AM and NS worked on the concept, inquiry, and validation. GY, NJ, and DC worked on resources and writing–original draft. SD, JT, MK, and HA worked on review and editing, visualization, project management, and supervision. All authors contributed to the article and approved the submitted version.
