# Supplementary material for: Interaction studies of Serendipita indica and Zhihengliuella sp. ISTPL4 and their synergistic role in growth promotion in rice
Source: Front Plant Sci. 2023 May 24;14:1155715. doi: 10.3389/fpls.2023.1155715 (PMC10244739; doi:10.3389/fpls.2023.1155715)
Supplement: Supplementary file 1 [file DataSheet_1.docx]

***Supplementary Material***

**Interaction studies of *Serendipita indica* and *Zhihengliuella* sp. ISTPL4 and their synergistic role in growth promotion in rice**

1. **Supplementary figures and tables**


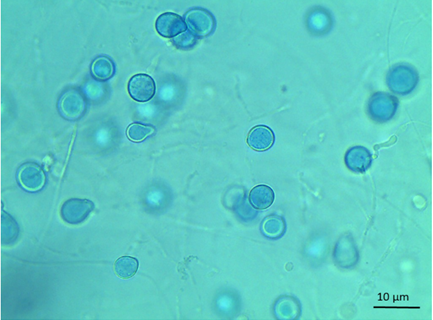

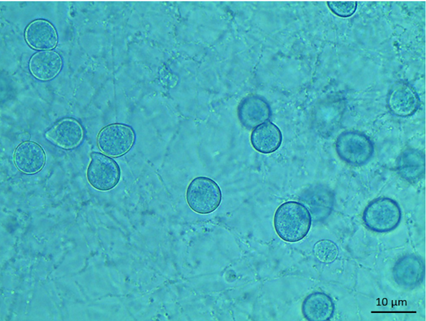


**(A)**

**(B)**

**Supplementary Figure 1**. Compound microscopic images **(A)** *S. indica* spores alone **(B)** *S. indica* spores in presence of *Z.* sp. ISTPL4

**Supplementary Table 1A.** Different concentrations of amino acids released by the individual culture of *S. indica* (S2) and *Z.* sp. ISTPL4 (S1) alone and co-culture of *S. indica* and *Z.* sp. ISTPL4 (S3) in dilution of 1:20

| **S. no** | **Amino acids (µM) (1:20)** | **S1 (Bacteria)** | **S2 (Fungus)** | **S3 (B+F)** |
| --- | --- | --- | --- | --- |
| **1** | Alanine | 30.986 ± 0.107 | 11.406 ± 0.26 | 25.487 ± 0.04 |
| **2** | Serine | 6.923 ± 0.05 | 3.440 ± 0.03 | 0.764 ± 0.001 |
| **3** | Valine | 1.729 ± 0.10 | 0.599 ± 0.01 | 1.257± 0.12 |
| **4** | Proline | 4.471± 0.02 | 1.360 ± 0.05 | 2.271± 0.31 |
| **5** | Threonine | 5.497± 0.02 | 2.114 ± 0.025 | 3.346± 0.17 |
| **6** | Isoleucine | 1.345 ± 0.01 | 0.605 ± 0.001 | 1.206 ± 0.20 |
| **7** | Glutamic acid | 15.126 ± 0.10 | 6.774± 0.02 | 11.511± 0.17 |
| **8** | Histidine | 2.807 ± 0.01 | 1.155 ± 0.01 | 2.066 ± 0.20 |
| **9** | Phenylalanine | 1.732 ± 0.01 | 0.787± 0.01 | 1.630 ± 0.21 |
| **10** | Arginine | 4.339 ± 0.12 | 1.796 ± 0.1 | 3.786± 0.20 |
| **11** | Tyrosine | 0.581 ± 0.15 | 0.335± 0.02 | 0.545± 0.14 |
| **12** | Tryptophan | 0.445 ± 0.03 | 0.193± 0.03 | 0.429± 0.01 |
| **13** | Glutamine | 6.607 ± 0.2 | 3.501±0.05 | 1.100± 0.05 |
| **14** | Lysine | 5.173± 0.11 | 2.361± 0.003 | 0.536 ± 0.002 |

**Supplementary Table 1B**. Different concentrations of amino acids released by the individual culture of *S. indica* (S2) and *Z.* sp. ISTPL4 (S1) alone and co-culture of *S. indica* and *Z.* sp. ISTPL4 (S3)

| **S. no** | **Amino acids (µmol/mg) dry weight:**  **(Volume 1ml)** | **S1 (Bacteria)**  **25mg** | **S2 (Fungus)**  **25mg** | **S3 (B+F)**  **25mg** |
| --- | --- | --- | --- | --- |
| **1** | Alanine | 1.239±0.56 | 0.456±0.025 | 1.019±0.074 |
| **2** | Serine | 0.277±0.025 | 0.138±0.0012 | 0.031±0.045 |
| **3** | Valine | 0.069±0.0012 | 0.024±0.011 | 0.050±0.046 |
| **4** | Proline | 0.179± 0.0032 | 0.054±0.0032 | 0.091±0.051 |
| **5** | Threonine | 0.220±0.065 | 0.085±0.0063 | 0.134±0.065 |
| **6** | Isoleucine | 0.054±0.0062 | 0.024±0.0065 | 0.048±0.063 |
| **7** | Glutamic acid | 0.605±0.0012 | 0.271±0.0046 | 0.460±0.078 |
| **8** | Histidine | 0.112±0.021 | 0.046±0.0085 | 0.083±0.071 |
| **9** | Phenylalanine | 0.069±0.18 | 0.031±0.0084 | 0.065±0.079 |
| **10** | Arginine | 0.174±0.056 | 0.072±0.0087 | 0.151±0.052 |
| **11** | Tyrosine | 0.023±0.046 | 0.013±0.008 | 0.022±0.012 |
| **12** | Tryptophan | 0.018±0.039 | 0.008±0.051 | 0.017±0.001 |
| **13** | Glutamine | 0.264±0.007 | 0.140±0.0058 | 0.044±0.003 |
| **14** | Lysine | 0.207±0.085 | 0.094±0.0095 | 0.021±0.001 |
